# Influence of Body Mass Index, Cancer Type and Treatment on Long-Term Metabolic and Liver Outcomes in Childhood Cancer Survivors

**DOI:** 10.3390/jcm11030878

**Published:** 2022-02-07

**Authors:** Agostino Milluzzo, Lucia Manuella, Emanuela Cannata, Giovanna Russo, Sandro La Vignera, Francesco Purrello, Andrea Di Cataldo, Laura Sciacca

**Affiliations:** 1 Department of Clinical and Experimental Medicine, Endocrinology, University of Catania Medical School, 95122 Catania, Italy; agomil@alice.it (A.M.); luciamanuella3@gmail.com (L.M.); 2 Department of Medical and Surgical Sciences and Advanced Technologies “GF Ingrassia”, University of Catania, 95122 Catania, Italy; 3 Department of Clinical and Experimental Medicine, Paediatric Oncohematology Unit, University of Catania Medical School, 95122 Catania, Italy; e.cannata80@gmail.com (E.C.); diberuss@unict.it (G.R.); adicata@unict.it (A.D.C.); 4 Department of Clinical and Experimental Medicine, University of Catania, Policlinico “G. Rodolico”, 95123 Catania, Italy; sandrolavignera@unict.it; 5 Department of Clinical and Experimental Medicine, Internal Medicine, Garibaldi-Nesima Hospital, University of Catania, 95123 Catania, Italy; francesco.purrello@unict.it

**Keywords:** childhood cancer survivors, obesity, metabolic syndrome, insulin resistance, liver steatosis, leukemia, lymphoma, chemotherapy, radiotherapy, bone marrow transplantation

## Abstract

In the last decade, the survival of subjects affected by cancer in childhood has significantly improved. The increased lifespan of childhood cancer survivors (CCS) led to a greater risk for long-term, therapy-related morbidity. To identify the clinical predictors of metabolic adverse outcomes in CCS (average off-therapy period: 12 years), we recruited 126 survivors of different childhood cancers (86.5% hematological cancers) who received at least anticancer chemotherapy, consecutively approached during their annual oncohematological outpatient visit. At examination, anthropometric measures and cancer-related history were collected. Moreover, a fasting venous sample was carried out for measuring fasting plasma glucose and insulin, glycated hemoglobin, lipid panel, and transaminases. We calculated the indexes of insulin resistance (HOMA-IR, McAuley, and QUICKI) and secretion (HOMA-β), liver steatosis (Hepatic Steatosis Index) and fibrosis (FIB-4 and NAFLD fibrosis score), and visceral fat dysfunction (Visceral Adiposity Index). More than one-third of the subjects (37.3%) did not have normal weight, with 11.1% of them affected by obesity. At recruitment, obese subjects were at significantly higher risk for impaired fasting glucose, metabolic syndrome, visceral adipose dysfunction, and liver steatosis/fibrosis. Subjects who received bone marrow transplantation were prone to insulin resistance, while survivors of lymphoma presented a visceral adipose dysfunction These results suggest a carefully metabolic monitoring of CCS, particularly in subgroups at higher risk, to early detect these conditions, promptly begin therapeutic interventions, and mitigate the dysmetabolic-related health burden.

## 1. Introduction

Cancers affecting pediatric and adolescent age represent 1–2% of all cancer [1]. Leukemia is the most common (33% of childhood tumors), followed by brain tumors, lymphomas, neuroblastoma, bone tumors (osteosarcoma and Ewing’s sarcoma), hepatoblastoma, Wilms’ tumor, rhabdomyosarcoma, and retinoblastoma [1]. Progress in research has led, in the last decade, to a significant improvement of the prognosis of children and adolescents with malignant tumors, whose 5-year survival rate reaches 90% in developed countries [2]. Although the anticancer therapeutic protocols have also been improved in terms of toxicity, the high survival rate and lifespan of childhood cancer survivors (CCS) led to a greater risk for long-term, treatment-related morbidity: cardiac damage (heart failure and coronary and valvular diseases), dysmetabolic conditions, bone loss, neuropathy, gonadal failure, infertility, sexual dysfunction, chronic pain and fatigue, insomnia, and accelerated aging [3,4,5,6,7,8]. Moreover, adult CCS have a significant decline in functional status, mental health, limitations on activity, and a poorer general health [3,4,6,9,10]. In particular, endocrine diseases (pituitary, thyroid, gonads, bone, and metabolic disorders), observed in 20–50% of adult CCS, are the second most frequent group of complications of these subjects [11]. The risk of long-term metabolic dysfunction in CCS depends by multiple factors, related to both treatment exposures and individual host factors. Firstly, anticancer therapies induce long-term insulin resistance (IR) and hyperinsulinemia, affecting carbohydrate and lipid metabolism. In fact, both chemotherapy and radiotherapy cause the release of inflammatory molecules (e.g., IL-6, IL-1β, and TNF-a) and the activation of macrophages that contribute to the development of IR, mainly responsible for the long-term onset of metabolic changes [7,9]. In addition, glucocorticoids, often used in the different stages of treatment, contribute to the accumulation of body fat and increase of insulin resistance, favoring the occurrence of dysmetabolic conditions [9,12].

The interference on insulin signaling and consequent insulin resistance due to the release of IL-6 and TNF-α induced by cancer therapy could also affect liver condition, increasing the risk of non-alcoholic fatty liver disease (NAFLD) and steatohepatitis (NASH), and, in later stages, leading to liver fibrosis/cirrhosis [13,14]. 

Secondly, it has been observed that CCS often have an increased dietary intake and a low level of physical exercise, resulting in an increased risk for obesity, insulin resistance, diabetes mellitus (DM), dyslipidemia, and metabolic syndrome [3,4]. In this cross-sectional study, we assessed the long-term metabolic outcomes of CCS who underwent chemotherapy, irradiation, and/or bone marrow transplantation (BMT).

## 2. Materials and Methods

### 2.1. Study Population

We enrolled 126 CCS, all disease free for at least 5 years, aged between 7 and 42 years at recruitment. These were consecutively approached during their annual, ordinary, outpatient follow-up visits at the Pediatric Oncohematology Unit of the University Hospital “Policlinico Rodolico-San Marco” in Catania, Italy, between March 2018 and December 2019. At cancer diagnosis, all subjects received at least anticancer chemotherapy, according to the protocols of the Italian Association of Pediatric Haematology and Oncology (AIEOP). Subjects treated with surgery were excluded.

For all subjects the following data were collected reviewing medical records: demographic characteristics, medical history, cancer-specific history and related therapies, smoking habit, physical exercise, and first-degree familiarity for DM, dyslipidemia, and hypertension. 

At examination, weight, height, body mass index (BMI), waist circumference (WC), and systolic (SBP) and diastolic (DBP) blood pressure were measured. BMI was calculated by dividing the weight in kilograms by height in square meters (kg/m^2^), to classify subjects as normal weight (BMI 18.5–24.9 kg/m^2^ or, for pediatric age, ≤85th percentile), overweight (BMI 25.0–29.9 kg/m^2^ or, for pediatric age, between 85th−97th percentile) or obese (BMI ≥ 30 kg/m^2^ or, for pediatric age, >97th) [15,16]. WC was measured at the iliac crest and considered abnormal when >88 cm in women and >102 cm in men or when >80 cm in women and >94 cm in men, according to the National Cholesterol Education Program Adult Treatment Panel III (NCEP ATP III) or International Diabetes Federation (IDF) criteria, respectively [15]. For pediatric age, WC was considered abnormal when >90th percentile [17]. Blood pressure was measured on the right arm with an auscultatory sphygmomanometer [18]. Hypertension was defined as SBP values ≥140 mmHg and/or DBP values ≥90 mmHg or, for pediatric age, when >90th percentile [16,18]. The metabolic syndrome was defined according to the National Cholesterol Education Program/Adult Treatment Panel III [19]. The protocol was approved by the internal Institutional Review Board (ethical approval code: 01/2018).

The 126 enrolled subjects were divided on the basis of the following characteristics:

− BMI classification: normal weight, overweight or obese;
− Anticancer treatments: only chemotherapy (OC), chemotherapy plus radiotherapy (CR), or bone marrow transplantation (BMT);
− Type of tumor: leukemia, lymphomas, or solid cancers.

Written informed consent was obtained from all participants in the study and their parents. The study was conducted in accordance with the ethical principles of the Declaration of Helsinki and its later amendments. 

### 2.2. Metabolic and Biochemical Assay

At recruitment, all subjects underwent a 12-h fasting venous sample to measure fasting plasma glucose (FPG) (enzymatic glucose–hexokinase method), fasting plasma insulinemia (FPI) (chemiluminescent microparticle immunoassay), and glycated hemoglobin (HbA1c) (high-performance liquid chromatography of the gradient ionic exchange). Criteria for glucose impairment diagnosis (impaired fasting glucose (IFG), impaired glucose tolerance (IGT), and DM) followed the American Diabetes Association guidelines [20]. Total cholesterol, HDL cholesterol, and triglycerides were assessed by enzymatic methods, and LDL cholesterol concentration was calculated with the Friedewald formula (cholesterol total-HDL-triglycerides/5) if triglycerides value were lower than 400 mg/dL [21]. The assessment of aspartate transaminase (AST) and alanine aminotransferase (ALT) was performed by auto-analyzer laboratory methods. In male subjects, the measurement of LH, FSH, and Total Testosterone (TT) was performed on blood samples by electrochemiluminescence (ECLIA).

### 2.3. Indexes of Insulin Resistance and Insulin Secretion

To estimate the level of insulin resistance, we calculated the Homeostasis Model Assessment of IR (HOMA-IR), the McAuley index, and the Quantitative Insulin Sensitivity Check Index (QUICKI). HOMA-IR was obtained according to the following formula: fasting plasma insulin (FPI) (μU/mL) × fasting plasma glucose (FPG) (mmol/L)/22.5 [16]. The McAuley index was calculated as follows: exp [2.63–0.28 log FPI (μU/mL)–0.31 log fasting triglycerides (mmol/L)] [17]. QUICKI was calculated with the formula: 1/[log FPI (μU/mL) + log FPG (mg/dL)] [18]. Subjects were considered as insulin resistant for values of HOMA-IR above 2.5, McAuley Index and QUICKI below 5.8 and 0.339, respectively [22,23,24]. 

The pancreatic β-cell function was estimated by the HOMA-β: FPI concentration (μU/mL) × 20/FPG (mmol/L) − 3.5 [22,24,25].

### 2.4. NAFLD and Liver Fibrosis

The presence of NAFLD was non-invasively assessed calculating the Hepatic Steatosis Index (HSI) according to the following formula: [8 × ALT (U/L)/AST(U/L) ratio + BMI (+2, if female; +2, if DM)] [26]. Values of HSI < 30.0 ruled out NAFLD with a sensitivity of 93.1%, whereas HSI > 36.0 defined NAFLD with a specificity of 92.4% [26]. 

To define liver fibrosis, we relied on simple and non-invasive tools based on serum biomarkers. In particular, the fibrosis-4 (FIB-4) and the NAFLD fibrosis score (NFS) are considered the most accurate for excluding advanced fibrosis, with negative predictive values above 90% [27,28,29]. NAFLD fibrosis score was calculated using the formula: −1.675 + 0.037 × age (years) + 0.094 × BMI (kg/m^2^) +1.13 × IFG/DM (yes = 1, no = 0) + 0.99 × AST (U/L)/ALT(U/L) ratio-0.013 × platelet count (10^9^/L)-0.66 × albumin (g/dL) [25]. The low cut-off of −1.455 excluded with high accuracy (93%) an advanced fibrosis, while the high cut-off of 0.676 has a 90% positive predictive value [29,30]. The FIB−4 score consists of four variables (age, ALT, AST, and platelet count) and was calculated according to the formula: [age (years) × AST (U/L)]/[platelets (10^9^) × √ALT (U/L)] [26,31]. An upper threshold of 3.25 and a lower threshold of 1.3 were adopted to define and exclude advanced fibrosis, respectively [27].

### 2.5. Visceral Adipose Function and Cardio-Metabolic Risk

The Visceral Adiposity Index (VAI) was calculated to investigate fat distribution and function and the cardio-metabolic risk related to visceral fat dysfunction [32,33]. VAI is a model based on simple parameters and calculated according the following gender-specific formulas [34]:

− Females: [WC (cm)/36.58 + (1.89 × BMI (kg/m^2^)] × (triglycerides (mg/dL)/0.81) × (1.52/HDL (mg/dL));
− Males: [WC (cm)/39.68 + (1.88 × BMI (kg/m^2^)] × (triglycerides (mg/dL)/1.03) × (1.31/HDL (mg/dL)).

As indicated by Amato et al., we adopted different age-specific cut-off values to define the adipose tissue dysfunction: age < 30 years, 2.53; age ≥ 30 < 42 years, 2.24; age ≥ 42 < 52 years, 1.93; age ≥ 52 < 66 years, 1.94; ≥66 years, 2.01 [34,35]. 

### 2.6. Statistical Analysis

The distribution of continuous variables was analyzed by using Shapiro–Wilk with Lilliefors significance correction and Kolmogorov–Smirnov tests. Graphic analyses of histogram and Q-Q normality graph and the asymmetry/standard error or kurtosis/standard error ratio supported the exploration of the distribution of continuous variables. 

Continuous, normally distributed variables were presented as mean ± standard deviation (SD) and compared by using the univariate analysis of variance (ANOVA). F-test was preliminarily performed to examine the homogeneity of the variance. Continuous, not normally distributed variables were presented as median and interquartile range (IQR) and compared with Kruskal–Wallis tests. Linear regression models were performed to evaluate the influence of possible confounders in the comparison of continuous variables.

The prevalence of dichotomous outcomes was expressed as a number and percentage. Yates’ Chi-squared or Fisher exact test was applied to detect percentage difference between groups. Multivariable logistic regression models have been created to consider the interference of possible confounders on each outcome occurrence. We calculated the adjusted odds ratios (OR) of the event after correcting for potential confounders. A two-sided *p* value < 0.05 was considered statistically significant. 

Statistical analyses were performed using IBM SPSS Statistics software version 20 (IBM Corp., Armonk, NY, USA).

## 3. Results

The age at cancer diagnosis of the 126 CCS, 66 males and 60 females, was 7.3 ± 4.6 years, while the average age at recruitment and off-therapy period were 20.4 ± 6.7 and 11.9 ± 6.8 years, respectively (Table 1).

**Figure 1:**
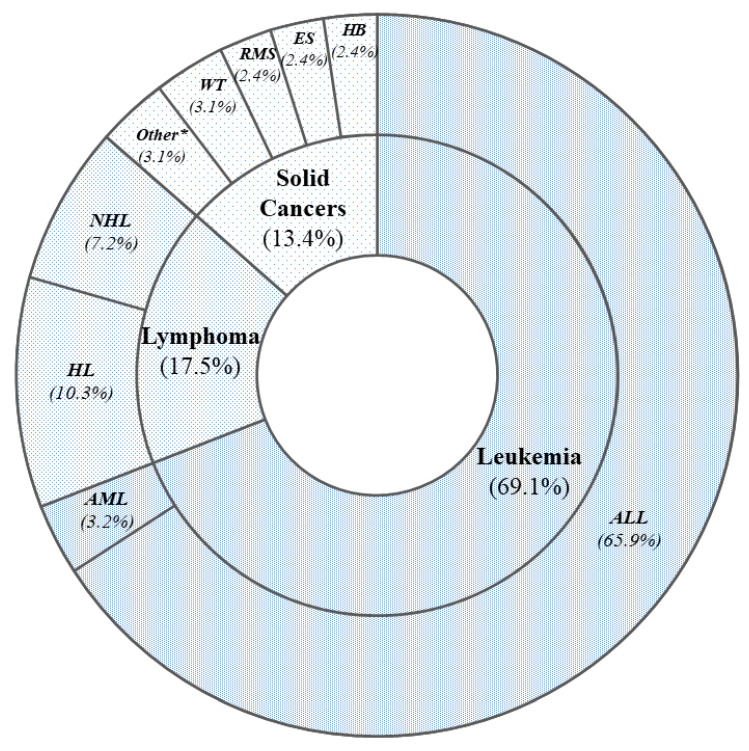
Distribution of the different cancer types in the whole cohort expressed in percent of total cancer cases. * In this group are included cancer types found in just one subject: retinoblastoma, neuroblastoma, germinal testicular cancer, and malignant peripheral nerve sheath cancer. Abbreviations: ALL, acute lymphoblastic leukemia; AML, acute myeloid leukemia; HL, Hodgkin lymphoma; NHL, non-Hodgkin lymphoma; WT, Wilms tumor; RMS, rhabdomyosarcoma; ES, Ewing sarcoma; HB, hepatoblastoma.

Most enrolled subjects (86.5%) were survivors of hematological cancers: acute lymphoblastic leukemia (ALL) (*n* = 83), acute myeloid leukemia (AML) (*n* = 4), Hodgkin lymphoma (HL) (*n* = 9), and non-Hodgkin lymphoma (NHL) (*n* = 13) (Figure 1). Wilms tumor (WT) (*n* = 4), rhabdomyosarcoma (*n* = 3), hepatoblastoma (*n* = 3), and Ewing sarcoma (*n* = 3) were the most represented solid cancers (Figure 1).

**Figure 2:**
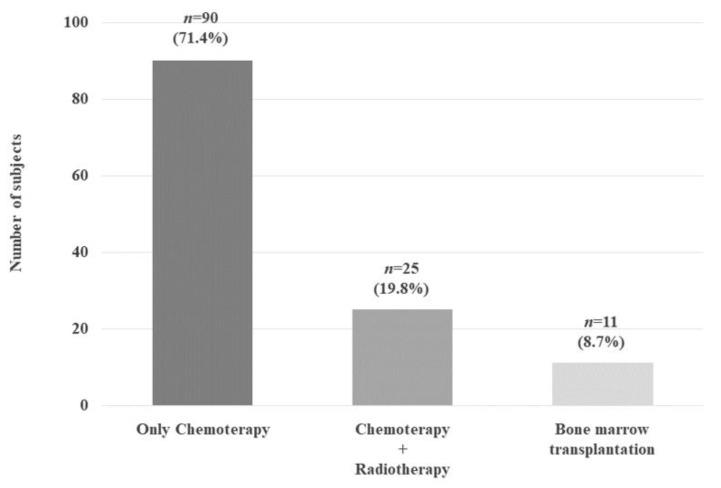
Distribution of the different anticancer therapies in the whole cohort expressed as the number of patients and in percent of total cancer cases.

Out of the 126 subjects (71.4%), 90 received only chemotherapy, while 25 subjects (19.8%) received both chemotherapy and radiotherapy. In 11 subjects (8.7%), BMT was performed (Figure 2). Although 12 subjects, during the follow-up, received further treatments due to disease relapse, at recruitment, they had been disease-free for at least 5 years.

### 3.1. Anthropometric, Clinical, and Metabolic Parameters

**Figure 3:**
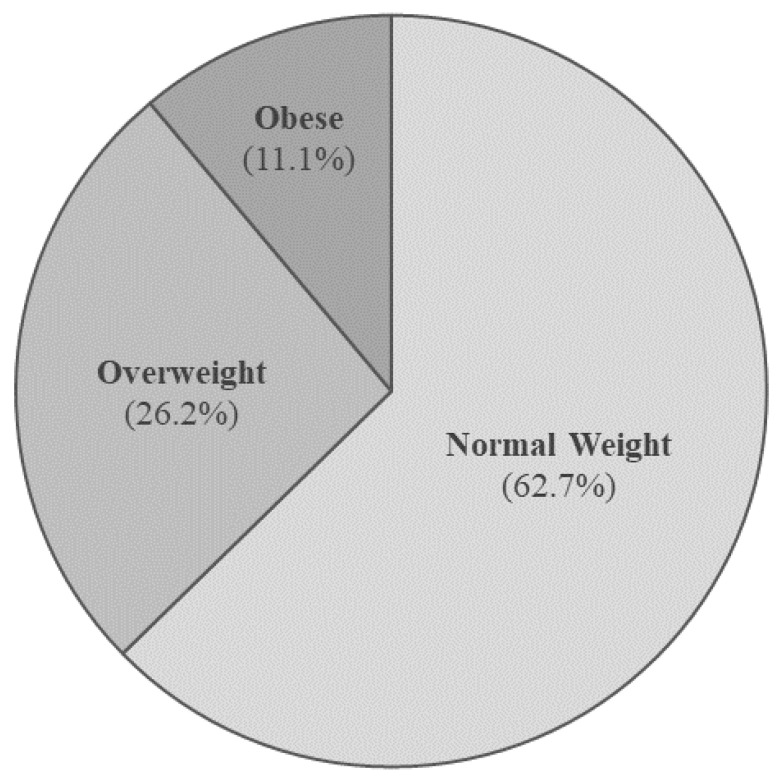
Distribution of subjects according to BMI expressed in percent of total cancer cases.

More than one-third of the subjects (37.3%) did not have normal weight, with 11.1% of them affected by obesity (Figure 3). An above normal WC was found in 27.8% and 53.2% of the cohort (according to NCEP ATP III and IDF criteria, respectively), and in all the obese subjects, whose WC was significantly higher vs. overweight and normal weight subjects (114.2 ± 7.1 vs. 95.7 ± 6.0 vs. 80.8 ± 9.0 cm, respectively; *p* < 0.001) (Table 2).

Subjects with previous leukemia, compared to both lymphoma and solid cancer groups, had a significantly lower prevalence (*p* < 0.05) of above normal WC (NCEP ATP III criteria: 21.8% vs. 40.9% vs. 43.8%, respectively; IDF criteria: 47.1% vs. 59.1% vs. 75.0%, respectively) and a trend for a lower BMI (23.5 ± 4.6 vs. 25.5 ± 5.5 vs. 24.4 ± 3.6 kg/m^2^, respectively; *p* = 0.16) (Table 3). These data have been confirmed by the multivariable analysis, considering the influence of physical activity, whose prevalence was significantly higher in CCS of lymphoma compared to leukemia and solid cancers (72.7% vs. 51.2% vs. 31.2%, respectively) (Table 3). 

As reported in Table 2 and Table 3, FPG, FPI, and HbA1c did not differ in the groups subdivided on the base of BMI or cancer type. At multivariable analysis, a trend, close to reaching statistical significance (*p* = 0.07), for an increased FPI was observed in subjects treated with BMT vs. both OC and CR groups (Table 4). None of the subjects were affected by DM, while five of them had an IFG. Obese subjects, compared to overweight and normal weight ones, had a significantly higher prevalence of IFG (14.3% vs. 6.1% vs. 1.2%, respectively; *p* = 0.05; OR 3.1). Moreover, subjects treated with BMT vs. OC and CR groups had a worst lipid profile, in particular a higher LDL level (129.4 ± 85.9 vs. 90.9 ± 72.2 vs. 76.6 ± 25.4 mg/dL, respectively; *p* = 0.21), although these differences did not reach a statistical significance, owing to the small number of cases, and should be evaluated in a larger cohort (Table 4).

The prevalence of metabolic syndrome did not differ between the groups based on cancer type and therapy type, but was significantly higher in obese vs. overweight and normal weight subjects (28% vs. 1.2% vs. 0.0%, respectively; *p* = 0.01; OR 7.4) at multivariable analysis (Table 2).

In obese subjects, compared to overweight and normal weight, we found significantly higher values of both SBP (133.8 ±17.4 vs. 117.9 ± 13.2 vs. 111 ± 11.7; respectively *p* < 0.001) and DBP (79.4 ± 17.6 vs. 69.3 ± 8.3 vs. 66 ± 8.8 respectively; *p* < 0.001) (Table 2).

### 3.2. Indexes of Insulin Resistance and Insulin Secretion

Subjects treated with BMT, compared to OC and CR, were more likely to develop insulin resistance, as suggested by the higher prevalence of abnormal HOMA-IR (75.0 vs. 13.6 vs. 33.3%, respectively; *p* = 0.03; OR 5.4) and QUICKI (75.0% vs. 22.7% vs. 33.3%, respectively; *p* = 0.05; OR 3.2) at multivariable analysis (Table 4). As illustrated in Table 2, obese subjects, compared to overweight and normal weight, had higher HOMA-IR (2.3) values, and a lower McAuley index (6.0), although these differences were not statistically significant. A similar trend was observed in subjects affected by lymphoma (HOMA-IR 2.7, HOMA-β 249.9, and McAuley index 5.4) (Table 3).

### 3.3. NAFLD and Liver Fibrosis

The index of fatty liver disease was worst in obese subjects (Table 2). In the obese group, compared to overweight and normal weight, we observed, at multivariable analysis, higher values for ALT (30.2 ± 15.7 vs. 29.3 ± 25.1 vs. 19.3 ± 15.9 U/L, respectively; *p* = 0.02), HSI (42.8 ± 5.5 vs. 39.0 ± 4.1 vs. 30.4 ± 4.6 U/L, respectively; *p* < 0.001), and NFS (−2.4 ± 0.9 vs. −3.8 ± 0.9 vs. −3.8 ± 1.0 U/L, respectively; *p* < 0.001) (Table 2). Moreover, almost all obese subjects and more than two-thirds of the overweight subjects had an HSI > 36. In these two groups, compared to normal weight subjects, a significantly higher prevalence of above normal HSI was observed (92.9% vs. 76.2% vs. 10.4%, respectively; *p* < 0.001; OR 34.1) (Table 2).

### 3.4. Visceral Adipose Function as a Surrogate of Cardio-Metabolic Risk

Subjects with VAI values above 2.53, if age < 30 years, and above 2.24, if age > 30 years, were considered affected by adipose tissue dysfunction. The prevalence of subjects with an above normal VAI was higher in obese subjects vs. overweight and normal weight (80.0% vs. 18.2% vs. 36.8%, respectively; *p* = 0.05; OR 2.6) (Table 2).

In about two-thirds of the subjects with previous lymphoma, an abnormal VAI was observed. These subjects had a significantly higher VAI vs. subjects with leukemia and solid cancers (5.9, IQR 2.4–12.3 vs. 2.1, IQR 1.9–3.8 vs. 1.3, IQR 0.9–2.2, respectively; *p* = 0.04) (Table 3).

In the BMT group, compared to OC and CR, we observed both the higher prevalence, although not significant, of subjects with abnormal VAI (60.0% vs. 30.4% vs. 42.9%, respectively; *p* = 0.13) and the higher VAI value (8.4, IQR 1.8–15.0 vs. 2.1, IQR 1.7–5.0 vs. 3.1 IQR 1.8–3.5; *p* = 0.14) (Table 4).

## 4. Discussion

This study evaluated the long-term metabolic effects of anticancer therapies in CCS young adults after an average off-therapy period of about 12 years. We analyzed whether BMI, cancer type, and anticancer treatment influence the development of different metabolic adverse outcomes, in order to identify subjects particularly at risk.

Several studies showed that CCS are prone to an excessive weight gain than their peers, due to glucocorticoids treatment, increased rate of hormonal dysfunction, excessive dietary intake, and a low level of physical exercise [4,5,36]. The prevalence of overweight and obesity in the previous published cohorts of CCS differs widely, according to age, follow-up duration, ethnicity, and social factors, as well as the prevalence of obesity in the general population of each country [37,38]. In our series, the prevalence of overweight (26.2%) and obesity (11.1%) was higher than indicated in the 2019 report of the Italian National Institute of Statistics (ISTAT) for the general population of the same age and country (15.8% and 3.0%, respectively), thus confirming the already reported tendency to overweight of childhood cancer survivors [39]. Obesity is a relevant health problem, particularly among the CCS frail population, representing a major risk factor for metabolic complications, such as insulin resistance, impaired glucose and lipid homeostasis, fatty liver, hypertension, and cardio-vascular disease [11]. For these reasons, in our cohort, we explored various biochemical metabolic variables and derived parameters to determine the prevalence in the recruited CCS of adipose tissue dysfunction, glucose and lipid impairment (impaired fasting glucose, diabetes mellitus), hypertension, and liver metabolic disease (NAFLD, NASH, and liver fibrosis/cirrhosis). Several studies reported that CCS are more prone to develop the metabolic syndrome, compared to the general population [11,40]. In accordance with previous evidence [11,40,41], we observed, in the obese group vs. normal weight and overweight, a significantly higher prevalence of metabolic syndrome (28.0%), and in particular, a significant higher WC, SBP, DPB (*p* < 0.001 for all), a higher prevalence of IFG (14.3%, *p* = 0.05), and a trend for a worse lipid profile (Table 2).

To estimate the cardio-metabolic risk related to visceral adiposity, we calculated the VAI, a sex-specific index based on WC, BMI, triglycerides, and HDL [33]. To our knowledge, no previous studies investigated the VAI in CCS. In our cohort, most of the obese subjects (80.0%) had a visceral adiposity dysfunction compared to 18.2% and 36.8% in overweight and normal weight subjects, respectively (*p* = 0.05) (Table 2). Nevertheless, our investigation did not clarify whether the observed increase prevalence of adipose dysfunction in obese CCS is directly related to fat tissue excess or to some other features concerning childhood cancer. Indeed, we also observed a very high VAI value both in survivors of lymphoma (5.9) and in subjects treated with BMT (8.4) (Table 3 and Table 4). A comparison with a control group of healthy subjects, matched for sex and age, could, in future research, explain these issues. Recent data demonstrated an increased risk of DM in CCS, in particular in subjects treated at a young age or exposed to radiotherapy [42,43]. In our series, none of the subjects were affected by DM. In five subjects, an IFG was diagnosed. This relatively low prevalence of overt glucose impairment could be explained by the young age at observation (20.4 years) and low off-therapy period (11.9 years) compared to previous studies [43] (Table 1). Nevertheless, we observed an early dysfunction of glucose homeostasis in parameters of insulin resistance and secretion: a clear trend for an increased insulin resistance, revealed by an increased HOMA-IR and a reduced McAuley index, was observed in subjects affected by obesity, in survivors of lymphoma, or in subjects treated with BMT (Table 1, Table 2 and Table 3). The percentage of physical inactivity and glucocorticoids use during cancer therapies, both known to impair glucose homeostasis, did not differ among subgroups according to BMI, cancer type, and treatment. In the BMT group, 75% of subjects had an above normal HOMA-IR, and this percentage was significantly higher than in the OC and CR groups, regardless of BMI (13.6% and 33.3%, respectively; *p* = 0.03) (Table 4). Few studies investigated the insulin resistance in CCS, observing an increased HOMA-IR in CCS already affected by DM [42]. Otherwise, in our investigation, we observed an insulin resistance also in CCS not affected by DM who had other risk factors for this disease. An early detection of forerunner of clinically manifest glucose impairment (IFG, IGT, and DM) is mandatory because it has been demonstrated, in cohorts of CCS, that the endothelial dysfunction and increase of cardio-metabolic risk precede the DM onset, and an immediate intervention on lifestyle (nutrition and physical exercise) could improve their cardio-metabolic status [44,45,46].

The negative effect of metabolic syndrome and insulin resistance on liver disease has been widely explained, and it is represented by a spectrum of conditions ranging from liver steatosis to NASH, and, in a later stage, liver fibrosis and cirrhosis [47,48,49]. No data are available on the risk of liver disease in CCS. Our study highlighted the bad impact of adiposity excess on liver steatosis, steatohepatitis, and fibrosis in CSS: Obese and overweight subjects had, compared to normal weight subjects, a significantly higher ALT and HSI value, indicating the onset of steatosis/steatohepatitis already in overweight subjects (Table 2). It is worth noting the very high prevalence of liver steatosis in obese and overweight groups (92.9% and 76.2%, respectively), while just 10.4% of normal weight subjects had an early liver disease (*p* < 0.001) (Table 2). These data became even more relevant considering the young age of the recruited subjects. Instead, a significant worsening of NFS was exclusively observed in obese but not in overweight nor normal weight subjects (*p* < 0.001) (Table 2). None of the recruited subjects, after an average off-therapy period of about 12 years, were affected by liver cirrhosis. To our knowledge, there are no data on the prevalence of advanced liver damage (fibrosis or cirrhosis) in subjects treated for cancer during childhood. In survivors of adult-onset cancers (median 30.0 years) who underwent BMT, Strasser and colleagues reported a cumulative incidence of liver cirrhosis of 0.6%, after 10 years since BMT, and 3.8%, after 20 years [50]. In our series, in subjects who underwent BMT, we found a trend for an increase of ALT and AST value, regardless of BMI, but no significant difference in terms of HSI, FIB-4, and NFS among the BMT, OC, and CR groups was observed (Table 4). (Table 3). In male subjects, LH, FSH, and TT were measured: no difference was found in the various subgroups (data not shown).

The present study shows some major limitations. First, the lack of a control group of metabolically healthy subjects, not affected by cancer in childhood, matched for sex and age. The second major limitation is the relatively small sample size. Since CCS were recruited in a single center, some of the differences observed could have reached statistical significance in a large cohort. Moreover, data concerning some environmental well-established risk factors for obesity and metabolic diseases (e.g., family socioeconomic status and parent education) were not available for our population. Finally, it could be relevant, in future research, to obtain data concerning the body composition and the glycemic response to oral glucose load as well as to integrate the index of liver steatosis/fibrosis calculated in our study with non-invasive (e.g., ultrasound and elastography) and, if clinically indicated, invasive (biopsy) liver instrumental exams.

The strength of our study is the real-world approach, featured by a cohort of subjects, treated in the same pediatric oncohematology unit, with uniform diagnostic and therapeutic protocols.

## 5. Conclusions

Our study indicated some clinical features that increase the likelihood of metabolic adverse outcomes in CCS. In particular, we observed that survivors of lymphoma, those previously treated with BMT, and those developing obesity during the follow-up are the most at risk for insulin resistance, liver steatosis or fibrosis, and visceral adipose dysfunction; the latter as a surrogate of increased cardio-metabolic risk. The prevalence of overweight and obesity was higher than in the general population of the same age and country. These results suggest a carefully metabolic monitoring in adults of CCS, particularly in subgroups at higher risk, both to early detect the long-term metabolic side effects of anticancer therapies and to begin preventive and therapeutic strategies. In this regard, metabolists, oncohematologists, nutritionists, and physical exercise experts should work in a coordinated “oncometabolic” team to achieve the best health outcomes for these frail subjects. Nevertheless, recommendations concerning screening modality, optimal timing of surveillance, and interventions are not available. In clinical practice, it would be useful to rely on protocols to manage metabolic screening of adult CCS. Further interventional studies with a larger sample and a long follow-up are mandatory to identify strategies to reduce the prevalence of obesity and the consequent metabolic burden in CCS.

## Figures and Tables

**Table 1:** Demographic, clinical, and metabolic characteristics of the 126 childhood cancer survivors.

Demographic and Anthropometric Features	
Male gender (%)	52.4
Age at recruitment (years)	20.4 ± 6.7
Age at cancer diagnosis (years)	7.3 ± 4.6
Follow-up since cancer diagnosis (years)	14.3 ± 6.8
Off-therapy period (years)	11.9 ± 6.8
BMI (kg/m^2^)	24.0 ± 4.6
Normal Weight (%)	62.7
Overweight (%)	26.2
Obese (%)	11.1
WC (cm)	88.4 ± 13.8
High WC (%)	
NCEP ATP III criteria	27.8
IDF criteria	53.2
Physical exercise (%)	53.2
Cigarette smoke (%)	17.5
**Blood pressure**	
SBP (mmHg)	115.3 ± 14.6
DBP (mmHg)	68.3 ± 10.8
**Metabolic syndrome (%)**	4.0
**Glucose and lipids metabolism**	
Glycemia (mg/dL)	86.2 ± 8.1
Insulinemia (μU/mL)	8.4 (7.4–10.3)
HbA1c (%)	5.3 (4.9–5.4)
DM (%)	0.0
IFG (%)	4.0
Cholesterol total (mg/dL)	168.0 ± 29.6
HDL (mg/dL)	49.8 ± 11.3
LDL (mg/dL)	94.3 ± 26.0
Triglycerides (mg/dL)	94.2 ± 68.4
**Insulin resistance and secretion**	
HOMA-IR	1.7 (1.7–2.2)
HOMA-IR > 2.5 (%)	24.1
HOMA-β	135.2 (91.4–242.7)
McAuley Index	8.5 (6.7–9.0)
McAuley Index < 5.8 (%)	12.0
QUICKI	0.4 (0.3–0.4)
QUICKI < 0.339 (%)	31.0
**Visceral adipose function**	
VAI	2.3 (1.9–4.3)
Above normal VAI (%)	37.1
**Liver steatosis and fibrosis**	
AST (U/L)	21.3 ± 13.1
ALT (U/L)	23.5 ± 19.1
HSI	34.7 ± 6.9
HSI ≥ 36 (%)	41.0
FIB-4	0.4 ± 0.2
FIB-4 > 3.25 (%)	0.0
NFS	−3.5 ± 1.1
NFS < −1.455 (%)	98.6
NFS > 0.676 (%)	0.0

Continuous variables are presented as mean ± SD or median [interquartile range]. Dichotomous variables are presented as a percentage (%) of the total subjects of each subgroup. Abbreviations: BMI, body mass index; WC, waist circumference; NCEP ATP III, National Cholesterol Education Program Adult Treatment Panel III; IDF, International Diabetes Federation; HbA1c, glycated hemoglobin; DM, Diabetes Mellitus; IFG, impaired fasting glucose; HDL, High-Density Lipoprotein; LDL, Low-Density Lipoprotein; SBP, Systolic Blood Pressure; DBP, Diastolic Blood Pressure; HOMA-IR, Homeostasis Model Assessment of Insulin-Resistance; QUICKI, Quantitative Insulin Sensitivity Check Index; VAI, Visceral Adiposity Index; AST, aspartate aminotransferase; ALT, alanine aminotransferase; HSI, Hepatic Steatosis Index; NFS, NAFLD fibrosis score.

**Table 2:** Demographic, clinical, and metabolic characteristics of the 126 childhood cancer survivors subdivided according to BMI.

	Normal Weight (*n* = 79)	Overweight (*n* = 33)	Obese (*n* = 14)	*p* *	OR
**Demographic and anthropometric features**					
Male gender (%)	47.5	51.5	78.6	0.10	
Age at recruitment (years)	20.7 ± 7.0	21.4 ± 5.4	23.8 ± 8.6	0.30	
Age at cancer diagnosis (years)	6.7 ± 4.7	7.6 ± 4.6	6.5 ± 4.0	0.56	
Follow-up since cancer diagnosis (years)	14.0 ± 6.7	13.8 ± 5.6	17.3 ± 9.3	0.21	
Off-therapy period (years)	11.9 ± 6.7	11.4 ± 6.03	14.5 ± 10.1	0.32	
BMI (kg/m^2^)	21.1 ± 2.5	27.4 ± 1.5	32.7 ± 2.1	**<0.001**	
WC (cm)	80.8 ± 9.0	95.7 ± 6.0	114.2 ± 7.1	**<0.001**	
High WC (%)					
NCEP ATP III criteria	8.8	42.4	100.0	**<0.001**	**11.8**
IDF criteria	31.2	84.8	100.0	**<0.001**	**10.2**
Physical exercise (%)	58.2	45.5	42.9	0.33	
Cigarette smoke (%)	21.5	6.1	21.4	0.13	
**Blood pressure**					
SBP (mmHg)	111 ± 11.7	117.9 ± 13.2	133.8 ±17.4	**0.001**	
DBP (mmHg)	66 ± 8.8	69.3 ± 8.3	79.4 ± 17.6	**0.001**	
**Metabolic syndrome (%)**	1.2	0.0	28.6	**0.01**	
**Glucose and lipids metabolism**					
Glycemia (mg/dL)	85.2 ± 7.2	86.4 ± 9.2	89.4 ± 8.9	0.46	
DM (%)	0.0	0.0	0.0	1.00	
IFG (%)	1.2	6.1	14.3	**0.05**	**3.1**
Cholesterol total (mg/dL)	164.0 ± 29.5	169.5 ± 35.6	177.4 ± 17.4	0.33	
HDL (mg/dL)	51.6 ± 11.5	48.1 ± 10.5	45 ± 12.4	0.07	
LDL (mg/dL)	95.0 ± 26.6	90.1 ± 30.2	102.3 ± 12.5	0.99	
Triglycerides (mg/dL)	90.1 ± 64.1	76.8 ± 44.3	125.6 ± 96.2	0.14	
**Insulin resistance and secretion**					
HOMA-IR >2.5 (%)	30.8	10.0	33.3	0.80	
McAuley Index < 5.8%	16.7	0.0	20.0	0.83	
QUICKI < 0.339 (%)	30.8	20.0	50.0	0.63	
**Visceral adipose function**					
Above normal VAI (%)	36.8	18.2	80.0	0.05	
**Liver steatosis and fibrosis**					
AST (U/L)	19.8 ± 14.5	21.3 ± 10.7	26.4 ± 10.7	0.13	
ALT (U/L)	19.3 ± 15.9	29.3 ± 25.1	30.2 ± 15.7	**0.02**	
HSI	30.4 ± 4.6	39.0 ± 4.1	42.8 ± 5.5	**<0.001**	
HSI ≥ 36 (%)	10.4	76.2	92.9	**<0.001**	**34.1**
FIB-4	0.4 ± 0.2	0.3 ± 0.2	0.5 ± 0.3	0.63	
FIB-4 > 3.25 (%)	0.0	0.0	0.0	1.00	
NFS	−3.8 ± 1.0	−3.8 ± 0.9	−2.4 ± 0.9	**0.001**	
NFS < −1.455 (%)	100	100	92.9	0.99	
NFS > 0.676 (%)	0.0	0.0	0.0	1.00	

Continuous variables are presented as mean ± SD or median [interquartile range]. Dichotomous variables are presented as a percentage (%) of the total subjects of each subgroup. Abbreviations: BMI, body mass index; WC, waist circumference; NCEP ATP III, National Cholesterol Education Program Adult Treatment Panel III; IDF, International Diabetes Federation; DM, Diabetes Mellitus; IFG, impaired fasting glucose; HDL, High-Density Lipoprotein; LDL, Low-Density Lipoprotein; SBP, Systolic Blood Pressure; DBP, Diastolic Blood Pressure; HOMA-IR, Homeostasis Model Assessment of Insulin-Resistance; QUICKI, Quantitative Insulin Sensitivity Check Index; VAI, Visceral Adiposity Index; AST, aspartate aminotransferase; ALT, alanine aminotransferase; HSI, Hepatic Steatosis Index; NFS, NAFLD fibrosis score. * p value in multivariable analysis models (logistic regression for dichotomous variables, linear regression for continuous variables) after adjusting for age at cancer diagnosis, age at recruitment, follow-up since cancer diagnosis, and physical exercise considering normal weight group as reference.

**Table 3:** Demographic, clinical, and metabolic characteristics of the 126 childhood cancer survivors subdivided according to the type of cancer.

	Leukemia (*n* = 87)	Lymphomas (*n* = 22)	Solid Cancers (*n* = 17)	*p* *
**Demographic and anthropometric features**				
Male gender (%)	55.2	54.5	25.0	0.08
Age at recruitment (years)	21.1 ± 7.2	23.0 ± 6.8	19.8 ± 4.3	0.33
Age at cancer diagnosis (years)	6.1 ± 4.3	10.6 ± 3.8	6.8 ± 5.0	**<0.001**
Follow-up since cancer diagnosis (years)	15.0 ± 7.3	12.1 ± 5.9	13.1 ± 4.3	0.16
Off-therapy period (years)	12.4 ± 7.5	11.1 ± 5.9	11.1 ± 5.9	0.64
BMI (kg/m^2^)	23.5 ± 4.6	25.5 ± 5.5	24.4 ± 3.6	0.16
WC (cm)	86.9 ± 13.6	94.3 ± 16.0	88.5 ± 9.5	0.19
High WC (%)				
NCEP ATP III criteria	21.8	40.9	43.8	0.04
IDF criteria	47.1	59.1	75.0	0.04
Physical exercise (%)	51.2	72.7	31.2	**0.03**
Cigarette smoke (%)	17.4	9.1	31.2	0.20
**Blood pressure**				
SBP (mmHg)	115.4 ± 15.2	118.8 ± 14.9	111.2 ± 9.7	0.63
DBP (mmHg)	67.6 ± 10.6	72.7 ± 12.6	67.2 ± 8.4	0.37
**Metabolic syndrome (%)**	4.6	4.5	0.0	0.63
**Glucose and lipids metabolism**				
Glycemia (mg/dL)	86.8 ± 8.4	84.2 ± 7.7	85.5 ± 6.9	0.56
Insulinemia (μU/mL)	8.0 (7.0–9.1)	13.9 (7.7–19.2)	9.4 (8.5–9.6)	0.24
HbA1c (%)	5.2 (4.9–5.5)	5.1 (4.4–5.3)	5.3 (5–5.5)	0.97
DM (%)	0.0	0.0	0.0	1.00
IFG (%)	4.6	4.6	0.0	0.62
Cholesterol total (mg/dL)	169.5 ± 29.2	161.0 ± 33.5	162.3 ± 24.3	0.67
HDL (mg/dL)	49.3 ± 10.7	45.6 ± 11.8	54.9 ± 13.4	0.18
LDL (mg/dL)	94.3 ± 65.4	124.3 ± 97.9	61.6 ± 24.0	0.67
Triglycerides (mg/dL)	97.2 ± 24.2	74.1 ± 38.1	96.1 ± 25.8	0.41
**Insulin resistance and secretion**				
HOMA-IR	1.7 (1.6–1.8)	2.7 (1–5–4.2)	2.2 (2.0–2.2)	0.65
HOMA-IR >2.5 (%)	25.0	42.9	0.0	0.37
HOMA-β	135.2 (88.2–192.6)	249.9 (151.3–325.1)	102.6 (90–119.8)	0.27
McAuley Index	8.7 (7.7–9.0)	5.4 (4.7–8.7)	8.4 (6.9–9.2)	0.55
McAuley Index < 5.8%	6.7	40.0	0.0	0.89
QUICKI	0.4 (0.3–0.4)	0.3 (0.3–0.4)	0.4 (0.3–0.3)	0.72
QUICKI < 0.339 (%)	25.0	57.1	20.0	0.90
**Visceral adipose function**				
VAI	2.1 (1.9–3.8)	5.9 (2.4–12.3)	1.3 (0.9–2.2)	**0.04**
Above normal VAI (%)	40.9	60.0	14.3	0.32
**Liver steatosis and fibrosis**				
AST (U/L)	22.3 ± 15.9	21.4 ± 5.2	17.4 ± 6.4	0.44
ALT (U/L)	25.7 ± 22. 3	22.0 ± 12.5	15.3 ± 7.8	0.11
HSI	34.7 ± 7.0	35.3 ± 7.5	33.4 ± 5.5	0.79
HSI ≥ 36 (%)	42.3	38.9	33.3	0.65
FIB-4	0.4 ± 0.2	0.5 ± 0.1	0.4 ± 0.1	0.58
FIB-4 > 3.25 (%)	0.0	0.0	0.0	1.00
NFS	−3.7 ± 1.0	−2.9 ± 1.0	−3.5 ± 1.5	**0.14**
NFS < −1.455 (%)	100.0	93.8	100.0	0.99
NFS > 0.676 (%)	0.0	0.0	0.0	1.00

Continuous variables are presented as mean ± SD or median [interquartile range]. Dichotomous variables are presented as a percentage (%) of the total subjects of each subgroup. Abbreviations: BMI, body mass index; WC, waist circumference; NCEP ATP III, National Cholesterol Education Program Adult Treatment Panel III; IDF, International Diabetes Federation; HbA1c, glycated hemoglobin; DM, Diabetes Mellitus; IFG, impaired fasting glucose; HDL, High-Density Lipoprotein; LDL, Low-Density Lipoprotein; SBP, Systolic Blood Pressure; DBP, Diastolic Blood Pressure; HOMA-IR, Homeostasis Model Assessment of Insulin-Resistance; QUICKI, Quantitative Insulin Sensitivity Check Index; VAI, Visceral Adiposity Index; AST, aspartate aminotransferase; ALT, alanine aminotransferase; HSI, Hepatic Steatosis Index; NFS, NAFLD fibrosis score. * *p* value in multivariable analysis models (logistic regression for dichotomous variables, linear regression for continuous variables) after adjusting for age at cancer diagnosis, age at recruitment, follow-up since cancer diagnosis, and physical exercise.

**Table 4:** Demographic, clinical, and metabolic characteristics of the 126 childhood cancer survivors subdivided according to the type of anticancer therapy.

	Chemotherapy (*n* = 90)	Chemo-Radio (*n* = 25)	Transplant (*n* = 11)	*p* *
**Demographic and anthropometric features**				
Male gender (%)	57.1	36	45.5	0.15
Age at recruitment (years)	20.4 ± 6.5	24.2 ± 6.0	21.7 ± 9.3	**0.03**
Age at cancer diagnosis (years)	6.2 ± 4.2	9.0 ± 5.0	8.0 ± 5.4	**0.02**
Follow-up since cancer diagnosis (years)	14.1 ± 6.1	15.1 ± 7.5	13.6 ± 10.3	0.79
Off-therapy period (years)	12.1 ± 6.5	13.2 ± 7.0	9.2 ± 10.1	0.28
BMI (kg/m2)	24.1 ± 4.8	23.9 ± 4.2	23.8 ± 4.6	0.72
WC (cm)	88.6 ± 14.0	88.7 ± 11.8	86.4 ± 16.9	0.48
High WC (%)				
NCEP ATP III criteria	26.4	36.0	18.2	0.89
IDF criteria	49.5	60.0	63.6	0.39
Physical exercise (%)	52.2	60.0	45.5	0.68
Cigarette smoke (%)	16.7	24.0	9.1	0.51
**Blood pressure**				
SBP (mmHg)	116.4 ± 15.1	113.9 ± 12.7	109.9 ± 13.5	**0.02**
DBP (mmHg)	68.5 ± 11.3	67.5 ± 9.5	69.1 ± 10.3	0.57
**Metabolic syndrome (%)**	4.4	0.0	9.1	0.96
**Glucose and lipids metabolism**				
Glycemia (mg/dL)	86.6 ± 7.7	86.9 ± 8.0	81.9 ± 11.0	0.31
Insulinemia (μU/mL)	8.7 (7.9–9.8)	6 (5.8–13.0)	17.7 (6.4–29.0)	0.07
HbA1c (%)	5.3 (4.9–5.5)	5.3 (4.2–5.4)	5.1 (4.8–5.3)	0.45
DM (%)	0.0	0.0	0.0	1.00
IFG (%)	3.3	4.0	9.1	0.56
Cholesterol total (mg/dL)	168.6 ± 32.4	158.2 ± 25.0	179.1 ± 21.2	0.66
HDL (mg/dL)	49.5 ± 10.5	53.3 ± 13.0	46.0 ± 13.8	0.74
LDL (mg/dL)	90.9 ± 72.2	76.6 ± 25.4	129.4 ± 85.9	0.21
Triglycerides (mg/dL)	90.6 ± 28.4	99.9 ± 27.0	100.9 ± 18.4	0.20
**Insulin resistance and secretion**				
HOMA-IR	1.9 (1.7–2.2)	1.3 (1.3–2.9)	3.4 (1.3–5.4)	0.01
HOMA-IR > 2.5 (%)	13.6	33.3	75.0	**0.03**
HOMA-β	136.3 (98.9–224.7)	77.1 (69.6–167.8)	462.2 (121.3–803.1)	0.08
McAuley Index	8.5 (6.9–9.0)	8.6 (6–8.8)	6.7 (3.8–9.6)	0.10
McAuley Index < 5.8%	11.1	0.0	25.0	0.99
QUICKI	0.3 (0.3–0.4)	0.4 (0.3–0.4)	0.3 (0.3–0.4)	0.07
QUICKI < 0.339 (%)	22.7	33.3	75.0	0.05
**Visceral adipose function**				
VAI	2.1 (1.7–5.0)	3.1 (1.8–3.5)	8.4 (1.8–15.0)	0.14
Above normal VAI (%)	30.4	42.9	60.0	0.13
**Liver steatosis and fibrosis**				
AST (U/L)	21.0 ± 14.3	18.8 ± 4.2	29.9 ± 16.1	0.23
ALT (U/L)	22.9 ± 17.1	19.4 ± 7.4	34.9 ± 37.1	0.10
HSI	34.5 ± 7.2	34.1 ± 6.1	37.5 ± 6.0	0.35
HSI ≥ 36 (%)	41.4	33.3	57.1	0.49
FIB-4	0.4 ± 0.2	0.4 ± 0.2	0.5 ± 0.4	0.9
FIB-4 > 3.25 (%)	0.0	0.0	0.0	1.00
NFS	−3.5 ± 1.1	−3.5 ± 0.9	−3.4 ± 1.3	0.70
NFS < −1.455 (%)	100.0	100.0	85.7	**0.99**
NFS > 0.676 (%)	0.0	0.0	0.0	1.00

Continuous variables are presented as mean ± SD or median [interquartile range]. Dichotomous variables are presented as a percentage (%) of the total subjects of each subgroup. Abbreviations: BMI, body mass index; WC, waist circumference; NCEP ATP III, National Cholesterol Education Program Adult Treatment Panel III; IDF, International Diabetes Federation; HbA1c, glycated hemoglobin; DM, Diabetes Mellitus; IFG, impaired fasting glucose; HDL, High-Density Lipoprotein; LDL, Low-Density Lipoprotein; SBP, Systolic Blood Pressure; DBP, Diastolic Blood Pressure; HOMA-IR, Homeostasis Model Assessment of Insulin-Resistance; QUICKI, Quantitative Insulin Sensitivity Check Index; VAI, Visceral Adiposity Index; AST, aspartate aminotransferase; ALT, alanine aminotransferase; HSI, Hepatic Steatosis Index; NFS, NAFLD fibrosis score. * *p* value in multivariable analysis models (logistic regression for dichotomous variables, linear regression for continuous variables) after adjusting for age at cancer diagnosis, age at recruitment, follow-up since cancer diagnosis, and physical exercise.

## Data Availability

Not applicable.

## References

[1] Oeffinger K.C., Mertens A.C., Sklar C.A., Yasui Y., Fears T., Stovall M., Vik T.A., Inskip P.D., Robison L.L. (2003). Obesity in Adult Survivors of Childhood Acute Lymphoblastic Leukemia: A Report from the Childhood Cancer Survivor Study. J. Clin. Oncol..

[2] Jemal A., Siegel R., Xu J., Ward E. (2010). Cancer Statistics, 2010. CA A Cancer J. Clin..

[3] Smith S.M., Link M.P., Effinger K.E. (2020). Minding the Gap for Survivors of Childhood Cancer. JAMA Oncol..

[4] Shapiro C.L. (2018). Cancer Survivorship. N. Engl. J. Med..

[5] Duca Y., di Cataldo A., Russo G., Cannata E., Burgio G., Compagnone M., Alamo A., Condorelli R.A., la Vignera S., Calogero A.E. (2019). Testicular Function of Childhood Cancer Survivors: Who Is Worse?. J. Clin. Med..

[6] Tumminia A., Milluzzo A., Cinti F., Parisi M., Tata F., Frasca F., Frittitta L., Vigneri R., Sciacca L. (2018). Abnormal 1-Hour Post-Load Glycemia during Pregnancy Impairs Post-Partum Metabolic Status: A Single-Center Experience. J. Endocrinol. Investig..

[7] Milluzzo A., Tumminia A., Vella V., Gianì F., Manzella L., Frittitta L., Belfiore A., Vigneri R., Sciacca L. (2019). Short-Term Adverse Effects of Anticancer Drugs in Patients with Type 2 Diabetes. J. Chemother..

[8] Milluzzo A., Barchitta M., Maugeri A., Agodi A., Sciacca L. (2022). Body mass index is related to short term retinal worsening in type 2 diabetes patients treated with anticancer drugs. Minerva Endocrinol..

[9] Milluzzo A., Vigneri P., Martorana F., Vigneri R., Sciacca L. (2020). Type 2 Diabetes and Cancer: Problems and Suggestions for Best Patient Management. Explor. Med..

[10] Barone R., Gulisano M., Cannata E., Padalino S., Saia F., Maugeri N., Pettinato F., lo Nigro L., Casabona A., Russo G. (2020). Self- and Parent-Reported Psychological Symptoms in Young Cancer Survivors and Control Peers: Results from a Clinical Center. J. Clin. Med..

[11] Van Santen H.M., Chemaitilly W., Meacham L.R., Tonorezos E.S., Mostoufi-Moab S. (2020). Endocrine Health in Childhood Cancer Survivors. Pediatr. Clin. N. Am..

[12] Roberts A., James J., Dhatariya K., Agarwal N., Brake J., Brooks C., Castro E., Gregory R., Higham C., Hobley L. (2018). Management of Hyperglycaemia and Steroid (Glucocorticoid) Therapy: A Guideline from the Joint British Diabetes Societies (JBDS) for Inpatient Care Group. Diabet. Med..

[13] Thatishetty A.V., Agresti N., O’Brien C.B. (2013). Chemotherapy-Induced Hepatotoxicity. Clin. Liver Dis..

[14] Buzzetti E., Pinzani M., Tsochatzis E.A. (2016). The Multiple-Hit Pathogenesis of Non-Alcoholic Fatty Liver Disease (NAFLD). Metabolism.

[15] Ross R., Neeland I.J., Yamashita S., Shai I., Seidell J., Magni P., Santos R.D., Arsenault B., Cuevas A., Hu F.B. (2020). Waist Circumference as a Vital Sign in Clinical Practice: A Consensus Statement from the IAS and ICCR Working Group on Visceral Obesity. Nat. Rev. Endocrinol..

[16] Valerio G., Maffeis C., Saggese G., Ambruzzi M.A., Balsamo A., Bellone S., Bergamini M., Bernasconi S., Bona G., Calcaterra V. (2018). Diagnosis, Treatment and Prevention of Pediatric Obesity: Consensus Position Statement of the Italian Society for Pediatric Endocrinology and Diabetology and the Italian Society of Pediatrics. Ital. J. Pediatr..

[17] Zimmet P., Alberti K.G.M., Kaufman F., Tajima N., Silink M., Arslanian S., Wong G., Bennett P., Shaw J., Caprio S. (2007). The Metabolic Syndrome in Children and Adolescents? An IDF Consensus Report. Pediatr. Diabetes.

[18] Williams B., Mancia G., Spiering W., Agabiti Rosei E., Azizi M., Burnier M., Clement D.L., Coca A., de Simone G., Dominiczak A. (2018). 2018 ESC/ESH Guidelines for the Management of Arterial Hypertension. Eur. Heart J..

[19] Expert Panel on Detection, Evaluation, and Treatment of High Blood Cholesterol in Adults (2001). Executive Summary of the Third Report of the National Cholesterol Education Program (NCEP) Expert Panel on Detection, Evaluation, and Treatment of High Blood Cholesterol in Adults (Adult Treatment Panel III). JAMA J. Am. Med. Assoc..

[20] American Diabetes Association (2021). 2. Classification and Diagnosis of Diabetes. Stand. Med. Care Diabetes—2021 Diabetes Care.

[21] Friedewald W.T., Levy R.I., Fredrickson D.S. (1972). Estimation of the Concentration of Low-Density Lipoprotein Cholesterol in Plasma, without Use of the Preparative Ultracentrifuge. Clin. Chem..

[22] Wallace T.M., Levy J.C., Matthews D.R. (2004). Use and Abuse of HOMA Modeling. Diabetes Care.

[23] McAuley K.A., Williams S.M., Mann J.I., Walker R.J., Lewis-Barned N.J., Temple L.A., Duncan A.W. (2001). Diagnosing Insulin Resistance in the General Population. Diabetes Care.

[24] Katz A., Nambi S.S., Mather K., Baron A.D., Follmann D.A., Sullivan G., Quon M.J. (2000). Quantitative Insulin Sensitivity Check Index: A Simple, Accurate Method for Assessing Insulin Sensitivity In Humans. J. Clin. Endocrinol. Metab..

[25] Reaven G.M. (2009). HOMA-Beta in the UKPDS and ADOPT. Is the Natural History of Type 2 Diabetes Characterised by a Progressive and Inexorable Loss of Insulin Secretory Function? Maybe? Maybe Not?. Diabetes Vasc. Dis. Res..

[26] Lee J.-H., Kim D., Kim H.J., Lee C.-H., Yang J.I., Kim W., Kim Y.J., Yoon J.-H., Cho S.-H., Sung M.-W. (2010). Hepatic Steatosis Index: A Simple Screening Tool Reflecting Nonalcoholic Fatty Liver Disease. Dig. Liver Dis..

[27] Castera L. (2020). Non-invasive Tests for Liver Fibrosis in NAFLD: Creating Pathways between Primary Healthcare and Liver Clinics. Liver Int..

[28] Xiao G., Zhu S., Xiao X., Yan L., Yang J., Wu G. (2017). Comparison of Laboratory Tests, Ultrasound, or Magnetic Resonance Elastography to Detect Fibrosis in Patients with Nonalcoholic Fatty Liver Disease: A Meta-analysis. Hepatology.

[29] Mahady S.E., Macaskill P., Craig J.C., Wong G.L.H., Chu W.C.W., Chan H.L.Y., George J., Wong V.W.S. (2017). Diagnostic Accuracy of Noninvasive Fibrosis Scores in a Population of Individuals With a Low Prevalence of Fibrosis. Clin. Gastroenterol. Hepatol..

[30] Angulo P., Hui J.M., Marchesini G., Bugianesi E., George J., Farrell G.C., Enders F., Saksena S., Burt A.D., Bida J.P. (2007). The NAFLD Fibrosis Score: A Noninvasive System That Identifies Liver Fibrosis in Patients with NAFLD. Hepatology.

[31] Shah A.G., Lydecker A., Murray K., Tetri B.N., Contos M.J., Sanyal A.J. (2009). Comparison of Noninvasive Markers of Fibrosis in Patients With Nonalcoholic Fatty Liver Disease. Clin. Gastroenterol. Hepatol..

[32] Amato M.C., Pizzolanti G., Torregrossa V., Misiano G., Milano S., Giordano C. (2014). Visceral Adiposity Index (VAI) Is Predictive of an Altered Adipokine Profile in Patients with Type 2 Diabetes. PLoS ONE.

[33] Amato M.C., Giordano C., Galia M., Criscimanna A., Vitabile S., Midiri M., Galluzzo A. (2010). Visceral Adiposity Index: A Reliable Indicator of Visceral Fat Function Associated with Cardiometabolic Risk. Diabetes Care.

[34] Amato M.C., Giordano C. (2014). Visceral Adiposity Index: An Indicator of Adipose Tissue Dysfunction. Int. J. Endocrinol..

[35] Amato M.C., Giordano C., Pitrone M., Galluzzo A. (2011). Cut-off Points of the Visceral Adiposity Index (VAI) Identifying a Visceral Adipose Dysfunction Associated with Cardiometabolic Risk in a Caucasian Sicilian Population. Lipids Health Dis..

[36] Van Waas M., Neggers S.J.C.M.M., Pieters R., van den Heuvel-Eibrink M.M. (2010). Components of the Metabolic Syndrome in 500 Adult Long-Term Survivors of Childhood Cancer. Ann. Oncol..

[37] Malhotra P., Kapoor G., Jain S., Jain S., Sharma A. (2021). Obesity and Sarcopenia in Survivors of Childhood Acute Lymphoblastic Leukemia. Indian Pediatr..

[38] Gunn H.M., Emilsson H., Gabriel M., Maguire A.M., Steinbeck K.S. (2016). Metabolic Health in Childhood Cancer Survivors: A Longitudinal Study in a Long-Term Follow-Up Clinic. J. Adolesc. Young Adult Oncol..

[39] Indagine Istat (2013). Aspetti Della Vita Quotidiana.

[40] Talvensaari K.K., Lanning M., Tapanainen P., Knip M. (1996). Long-Term Survivors of Childhood Cancer Have an Increased Risk of Manifesting the Metabolic Syndrome. J. Clin. Endocrinol. Metab..

[41] Guler E., Col N., Buyukcelik M., Balat A. (2018). Prevalence of Hypertension Determined by Ambulatory Blood Pressure Monitoring (ABPM) and Body Composition in Long-Term Survivors of Childhood Cancer. Pediatr. Hematol. Oncol..

[42] Friedman D.N., Tonorezos E.S., Cohen P. (2019). Diabetes and Metabolic Syndrome in Survivors of Childhood Cancer. Horm. Res. Paediatr..

[43] Meacham L.R., Sklar C.A., Li S., Liu Q., Gimpel N., Yasui Y., Whitton J.A., Stovall M., Robison L.L., Oeffinger K.C. (2009). Diabetes Mellitus in Long-Term Survivors of Childhood Cancer. Arch. Intern. Med..

[44] Davis N.L., Tolfrey K., Jenney M., Elson R., Stewart C., Moss A.D., Cornish J.M., Stevens M.C.G., Crowne E.C. (2020). Combined Resistance and Aerobic Exercise Intervention Improves Fitness, Insulin Resistance and Quality of Life in Survivors of Childhood Haemopoietic Stem Cell Transplantation with Total Body Irradiation. Pediatr. Blood Cancer.

[45] Cohen J.E., Wakefield C.E., Cohn R.J. (2016). Nutritional Interventions for Survivors of Childhood Cancer. Cochrane Database Syst. Rev..

[46] Giordano P., Muggeo P., Delvecchio M., Carbonara S., Romano A., Altomare M., Ricci G., Valente F., Zito A., Scicchitano P. (2017). Endothelial Dysfunction and Cardiovascular Risk Factors in Childhood Acute Lymphoblastic Leukemia Survivors. Int. J. Cardiol..

[47] Kawaguchi T., Tsutsumi T., Nakano D., Torimura T. (2021). MAFLD: Renovation of Clinical Practice and Disease Awareness of Fatty Liver. Hepatol. Res..

[48] Lonardo A., Mantovani A., Lugari S., Targher G. (2020). Epidemiology and Pathophysiology of the Association between NAFLD and Metabolically Healthy or Metabolically Unhealthy Obesity. Ann. Hepatol..

[49] Kim H., Lee D.S., An T.H., Park H.-J., Kim W.K., Bae K.-H., Oh K.-J. (2021). Metabolic Spectrum of Liver Failure in Type 2 Diabetes and Obesity: From NAFLD to NASH to HCC. Int. J. Mol. Sci..

[50] Strasser S.I., Sullivan K.M., Myerson D., Spurgeon C.L., Storer B., Schoch H.G., Murakami C.S., McDonald G.B. (1999). Cirrhosis of the Liver in Long-Term Marrow Transplant Survivors. Blood.

